# Leiomyoma of Kidney

**DOI:** 10.15586/jkcvhl.v10i2.264

**Published:** 2023-06-26

**Authors:** Vishnu Karayil R, Mini Bhaskarashenoy, Geetha Sukumaran

**Affiliations:** Department of Pathology, Government T D Medical College, Alappuzha, Kerala, India

**Keywords:** renal leiomyomas, tumors, wedge resection, kidney

## Abstract

Renal leiomyomas are rare benign mesenchymal tumors of kidney that affect adults of second to sixth decade. They can present as small asymptomatic multifocal lesions that are identified only in autopsy, or as large solitary lesions that cause pain and abdominal distention. Histomorphologically it appears exactly like its counterpart in other soft tissues. Differentiating renal leiomyoma from the lipid-poor angiomyolipoma is difficult by morphology, hence immunohistochemical studies are recommended. The case described is that of a female patient aged 74 years with a small solitary lesion in right kidney, who presented with history of pain and abdominal distention. She underwent wedge resection, histopathologically and immunohistochemically diagnosed as renal leiomyoma.

## Introduction

Renal leiomyoma is an uncommon benign mesenchymal tumor of the kidneys. Although smooth muscle tumors can occur in various sites, especially urinary bladder and uterine myometrium, renal lesions are unusual as normal kidney parenchyma have smooth muscles only in blood vessels.

The characteristic gross appearance aids in the diagnosis, however the histomorphology can cause a subjective bias toward more common differentials of similar picture.

## Case Presentation

We would like to report a case of right-sided renal leiomyoma.

A 74-year-old woman with type 2 diabetes and hypertension on medical treatment presented with abdominal pain. Radiological evaluation showed a focal kidney lesion and multiple uterine fibroids.

CECT over whole abdomen was done, where a 12 × 8 mm well-defined, hyperdense, well-enhancing lesion was noted in the interpolar region of the right kidney with no fat. Uterus appeared bulky with few fibroids, and both adnexa appeared normal. All other organs showed no significant pathology. From the above clinical and radiological findings, the possibilities considered were a small renal cell carcinoma or a lipid-poor angiomyolipoma of the right kidney. For histopathology confirmation, a right renal wedge resection was done. As the tumor was small, and the whole mass with a margin of normal kidney could be removed en-bloc, our team of urologists preferred a wedge resection over a radical nephrectomy. The renal mass and the adjacent perirenal fat were sent separately.

The renal mass consisted of a single whitish nodular tissue piece measuring 2 × 2 × 1.5 cm. The surface was smooth. Cut section showed a well-circumscribed, firm, grey–white whorled lesion measuring 1.4 × 1.3 × 1.5 cm. No cystic areas, areas of calcification, or necrosis were identified. Periphery showed a rim of renal tissue. The separately sent perirenal fat measured 4 × 3 × 2 cm, cutting through which showed no solid areas or lymph nodes.

**Figure 1:**
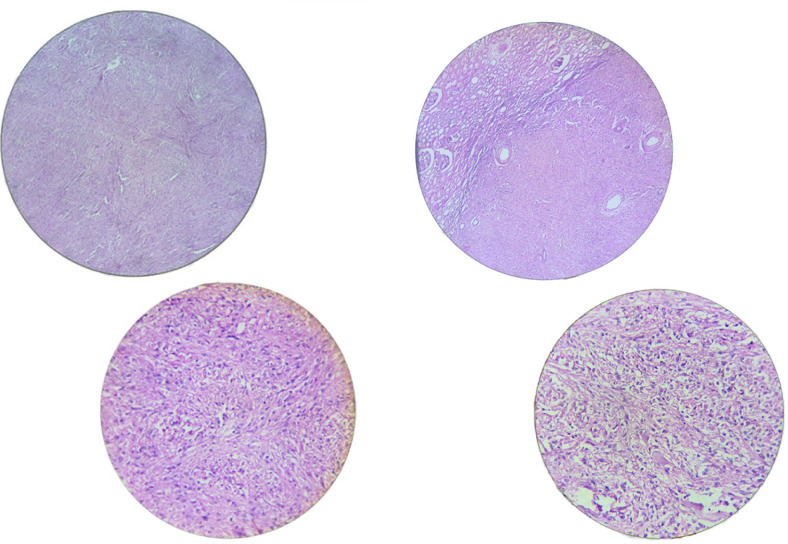
Immunohistochemical studies showing spindle cells were positive for smooth muscle actin and desmin, and negative for S-100 protein, HMB-45, CD34, and melan-A.

**Figure 2:**
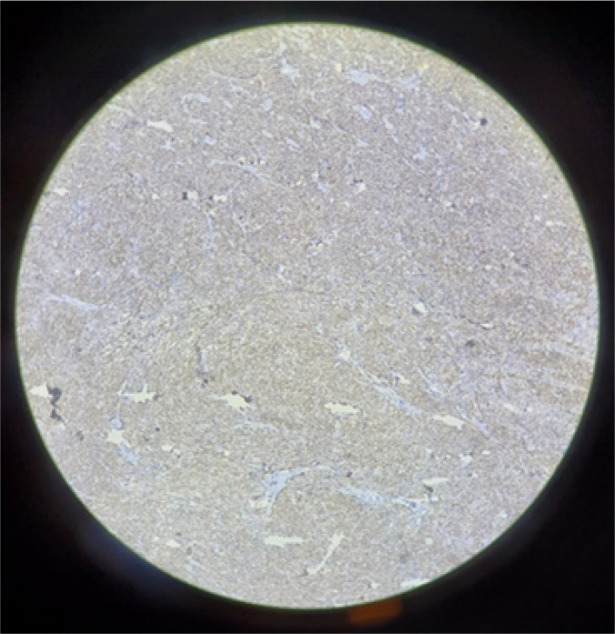
Spindle cells are positive for SMA.

**Figure 3:**
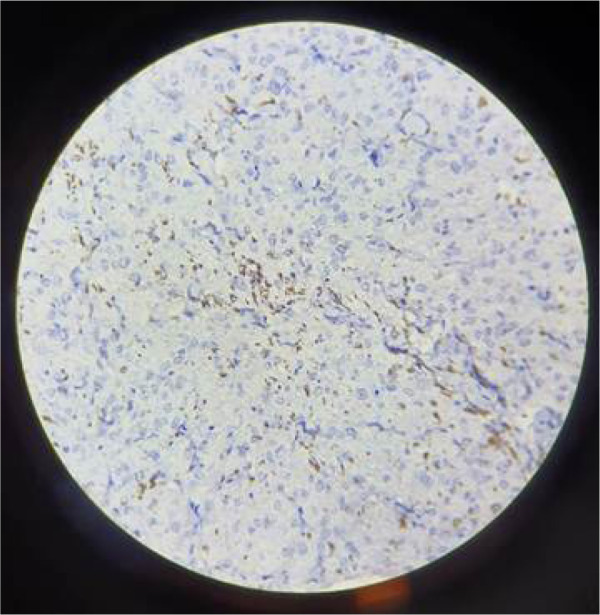
Spindle cells are positive for desmin.

**Figure 4:**
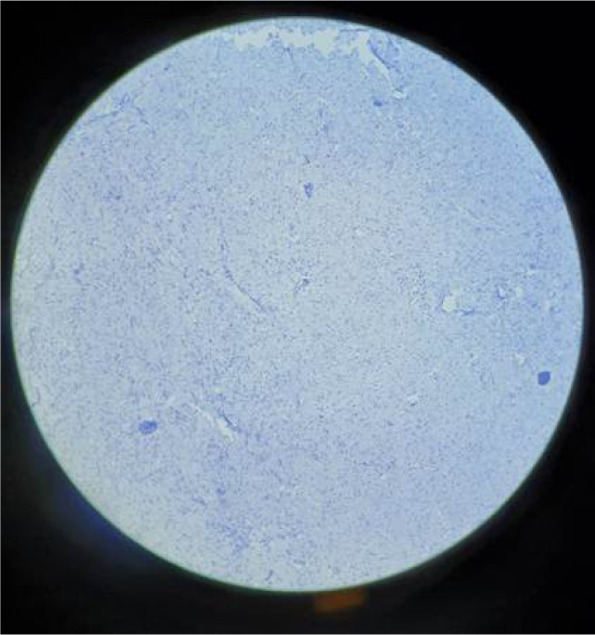
Spindle cells are negative for HMB45.

The entire circumference of the grey–white whorled lesion was sliced and embedded. Microscopic examination of the sections revealed renal tissue with a neoplasm composed of cells arranged in interlacing fascicles and bundles. Cells were long and spindly with blunt ends having moderate eosinophilic cytoplasm, elongated cigar-shaped bland nucleus with blunt ends. No atypia, mitosis, or necrosis was noted; however, scattered thin-walled vascular spaces were noted. Immunohistochemical studies were done which showed that the spindle cells were positive for smooth muscle actin and desmin, and negative for S-100 protein, HMB-45, CD34, and melan-A (Refer Figures 1–4).

The separate piece of perirenal fat was all embedded, and multiple sections were studied. No significant pathology was identified.

Histopathological and immunohistochemical findings confirmed the diagnosis of renal leiomyoma.

The patient was discharged later when all symptoms subsided, and the vitals were stable.. She continues on follow up every 6 months at our institution.

## Discussion

Renal leiomyomas are rare, benign mesenchymal tumors of the kidney arising from smooth muscle cells. It was first described in 1854 by Virchow (1). Leiomyomas occur commonly in urinary bladder, and they are extremely rare in kidneys or ureters. Renal leiomyomas comprise 0.3% of the overall nephrectomies worldwide (2).

With the very limited data from the reported cases, adults of second to sixth decade are more affected than children, with peak age of incidence at 42 years, and slight predominance in women (3). Over the years, the incidence of this disease has decreased substantially due to reclassification of an older described subset of tumors into HMB45-positive leiomyomatous angiomyolipoma. But with the advent of newer imaging techniques and autopsy techniques, small asymptomatic leiomyomas are being detected. Steiner et al. has classified these tumors into two: The ffirst group includes small asymptomatic tumors less than 5 mm, often multifocal and diagnosed incidentally in imaging studies or autopsy. The ssecond group includes large solitary symptomatic tumors, often presenting with abdominal pain and abdominal mass (3).

Grossly, these lesions are variably sized, ranging from less than 0.5 cm to as large as 57.5 cm. the hheaviest leiomyoma of the kidney reported to date weighed 37.2 kg. They are well-circumscribed, unencapsulated nodular firm masses. Cut surface tends to bulge out after fixation and is whitish whorled, with large tumors having peripheral reddish areas of peripheral vascularization. Cut surfaces tend to bulge out after fixation (3).

Microscopically, the tumor has the histomorphology of its counterparts in other soft tissues, a well-circumscribed neoplasm composed of cells arranged in long, broad interlacing fascicles. Cells are long fusiform spindled, with abundant eosinophilic cytoplasm and bland, elongated nucleus with blunt ends (so called cigar- or box-car-shaped). The tumor shows absence of mitotic figures, pleomorphism, hyperchromatism, or perilesional invasivity (3, 4, 5). Stroma is variably collagenized.

Immunohistochemically, the lesions are positive for vimentin, actin, smooth muscle myosin, desmin, laminin, and type IV collagen. The tumor cells are negative for low molecular weight keratin, c-Kit, and HMB45. The tumor has a very low Ki67 proliferation index (6).

The main differential diagnoses are leiomyosarcoma of kidney and lipid-poor angiomyolipoma. Leiomyosarcomas have areas of necrosis, features of cytological atypia, and a high Ki67 proliferation index. Angiomyolipomas have the characteristic vasculature, condensation of cells around the vessels, and stromal adipose tissue. But lipid-poor angiomyolipoma is a close differential diagnosis histologically.

Lipid-poor angiomyolipomas have the same histomorphology of renal leiomyoma and is comparatively more common. The only diagnostic aid is their positivity with HMB45 and c-Kit. Neither the positivity with SMA and desmin nor the negativity with S100 can differentiate between angiomyolipoma and leiomyoma. HMB45-negative lipid-poor angiomyolipomas have shown c-Kit positivity (4, 5).

The cell of origin is postulated to be from smooth muscles of the renal capsule, renal pelvis, or vascular smooth muscles around renal vasculature (4). Carpenter et al. has described the genetic predisposition in patients having aberrations involving chromosome 12q13. Apart from that, no significant genetic alterations have been described yet (3).

Treatment for renal leiomyoma is surgical excision and observation. The tumor has excellent prognosis following excision (5).

## Conclusion

In conclusion, we describe a case of renal leiomyoma in the right kidney. The clinico-radiological picture suspected a small renal cell carcinoma or-lipid poor angiomyolipoma. Even in histomorphology, the tumor very well can be confused with lipid-poor angiomyolipoma. So, immunohistochemical studies with SMA, desmin, HMB45, and c-Kit are recommended for the diagnosis.

## Regulatory Review

This study was determined to be exempt from regulatory review by the Institutional Review Board (IRB) as it involved only a retrospective review of chart and reports of a de-identified patient. No experimental interventions or invasive procedures were performed as part of this study, and there were no identifiable risks or harms to patients. As a result, the IRB determined that formal review was not necessary.
